# Supplementary material for: A Dual Coverage Monitoring of the Bile Acids Profile in the Liver–Gut Axis throughout the Whole Inflammation-Cancer Transformation Progressive: Reveal Hepatocellular Carcinoma Pathogenesis
Source: Int J Mol Sci. 2023 Feb 21;24(5):4258. doi: 10.3390/ijms24054258 (PMC10001964; doi:10.3390/ijms24054258)
Supplement: Supplementary file 1 [file ijms-24-04258-s001.zip › ijms-2189627-supplementary.pdf]

# Supporting Information

## Table of Contents


1. Supplemental Table S1. Standards used in this study.
2. Supplemental Table S2. List of the selectd MRM parameters, declustering potential (DP), entrance potential (EP), collision energy (CE), and cell exit potential (CXP) for each analyte.
3. Supplemental Table S3. Concentrations of analytes (μg/ml) in rat plasma, liver and intestinal contents samples from healthy control, hepatitis, cirrhosis, HCC and advanced HCC stage.(mean ± SD)
4. Supplemental Table S4. The classification results by BLDA.
5. Supplemental Table S5. 15 gene sets with FDR<25%, NES>1.5 and good significance (*P*-value<0.05) of GSEA enrichment analysis for patient samples which collected from TCGA database.
6. Supplemental Figure. S1. 15 gene sets with FDR<25%, NES>1.5 and good significance (*P*-value<0.05) of GSEA enrichment analysis for patient samples which collected from TCGA database. The order of the gene sets is arranged according to NES value (A-O).
7. Supplemental Figure. S2. BAAT expression is generally downregulated in human HCC tissues. Expression of BAAT in patients with HCC based on data from TCGA Liver Hepatocellular Carcinoma dataset.

**Table S1.** Standards used in this study.

| NO. | Analytes | Abbreviation | CAS number | Manufacturer |
|---|---|---|---|---|
| 1 | Cholic acid | CA | 81-25-4 | Yuanye Biology |
| 2 | Ursodeoxycholic acid | UDCA | 128-13-2 | Yuanye Biology |
| 3 | Chenodeoxycholic acid | CDCA | 474-25-9 | Yuanye Biology |
| 4 | Deoxycholic acid | DCA | 83-44-3 | Yuanye Biology |
| 5 | Lithocholic acid | LCA | 434-13-9 | Yuanye Biology |
| 6 | Glycocholic acid | GCA | 475-31-0 | Yuanye Biology |
| 7 | Glycoursodeoxycholic acid | GUDCA | 64480-66-6 | Yuanye Biology |
| 8 | Glycochenodeoxycholic acid | GCDCA | 640-79-9 | Yuanye Biology |
| 9 | Glycodeoxycholic acid | GDCA | 360-65-6 | Yuanye Biology |
| 10 | Glycolithocholic acid | GLCA | 474-74-8 | Yuanye Biology |
| 11 | Taurocholic acid | TCA | 81-24-3 | Yuanye Biology |
| 12 | Taurochenodeoxycholic acid | TCDCA | 516-35-8 | Yuanye Biology |
| 13 | Taurolithocholic Acid | TLCA | 6042-32-6 | Yuanye Biology |
| 14 | Taurodeoxycholic acid | TDCA | 1180-95-6 | Yuanye Biology |
| 15 | Tauroursodeoxycholic acid | TUDCA | 14605-22-2 | Yuanye Biology |
| 16 | Lithocholic-$d_4$ acid (IS) | LCA-$d_4$ | 83701-16-0 | Sigma-Aldrich |
| 17 | Chenodeoxycholic-$d_4$ acid (IS) | CDCA-$d_4$ | 99102-69-9 | Sigma-Aldrich |
| 18 | Deoxycholic-$d_4$ acid (IS) | DCA-$d_4$ | 112076-61-6 | Sigma-Aldrich |
| 19 | Glycochenodeoxycholic-$d_4$ acid (IS) | GCDCA-$d_4$ | 1201918-16-2 | Sigma-Aldrich |
| 20 | Cholic-$d_4$ acid (IS) | CA-$d_4$ | 116380-66-6 | Sigma-Aldrich |
| 21 | Glycodeoxycholic-$d_4$ acid (IS) | GDCA-$d_4$ | 1069132-37-1 | Sigma-Aldrich |
| 22 | Tauroursodeoxycholic acid-$d_4$ (IS) | TUDCA-$d_4$ | 2410279-94-4 | Sigma-Aldrich |

**Table S2.** List of the selected MRM parameters, declustering potential (DP), entrance potential (EP), collision energy (CE),
and cell exit potential (CXP) for each analyte.

| Analytes | Q1 Mass (Da) | Q3 Mass (Da) | DP (V) | CE (V) | EP (V) | CXP (V) |
|---|---|---|---|---|---|---|
| CA | 535.5 | 462.4 | 84.9 | 11.1 | 52.0 | 33.1 |
| UDCA | 519.6 | 446.5 | 97.9 | 12.1 | 57.9 | 24.1 |
| CDCA | 519.6 | 446.3 | 80.8 | 14.3 | 52.7 | 23.0 |
| DCA | 519.6 | 446.5 | 107.1 | 10.1 | 52.1 | 32.1 |
| LCA | 503.7 | 430.0 | 82.0 | 10.1 | 49.2 | 29.2 |
| GCA | 592.7 | 592.7 | 87.9 | 10.7 | 26.1 | 14.1 |
| GUDCA | 576.7 | 576.7 | 138.7 | 6.1 | 5.0 | 15.8 |
| GCDCA | 576.7 | 576.7 | 118.8 | 7.7 | 5.0 | 18.0 |
| GDCA | 576.7 | 576.7 | 80.9 | 10.0 | 5.0 | 23.3 |
| GLCA | 560.8 | 560.8 | 153.9 | 12.1 | 5.0 | 13.1 |
| TCA | 514.3 | 79.9 | -130.0 | -111.8 | -10.0 | -13.0 |

| | | | | | | |
|---|---|---|---|---|---|---|
| TCDCA | 498.3 | 79.9 | -128.3 | -103.7 | -10.0 | -13.8 |
| TLCA | 482.3 | 79.9 | -148.8 | -114.6 | -10.0 | -13.2 |
| TDCA | 498.3 | 79.9 | -150.0 | -109.1 | -10.0 | -13.0 |
| TUDCA | 498.3 | 79.9 | -146.2 | -113.7 | -10.0 | -13.1 |
| LCA-$d_4$ | 507.7 | 434.2 | 135.9 | 10.1 | 54.8 | 20.8 |
| CDCA-$d_4$ | 523.8 | 450.8 | 146.0 | 9.8 | 56.0 | 20.0 |
| DCA-$d_4$ | 523.7 | 450.3 | 129.1 | 6.8 | 54.8 | 15.9 |
| GCDCA-$d_4$ | 580.6 | 580.6 | 105.7 | 13.0 | 5.0 | 15.9 |
| CA-$d_4$ | 539.4 | 466.4 | 132.9 | 53.0 | 13.8 | 13.0 |
| GDCA-$d_4$ | 580.7 | 580.7 | 145.2 | 12.6 | 5.0 | 16.8 |
| TUDCA-$d_4$ | 502.3 | 79.9 | -144.8 | -123.0 | -10.0 | -13.7 |

**Table S3.** Concentrations of analytes (μg/ml) in rat plasma, liver and intestinal contents samples from healthy control, hepatitis, cirrhosis, HCC and advanced HCC stage.(mean ± SD).The beginning of "P-" represents bile acids in plasma, while the beginning of "L-" and "I-" represent bile acids in liver and intestinal contents.

| Analytes | Health contronl | Hepatitis | Cirrhosis | HCC | Advance HCC |
|---|---|---|---|---|---|
| P-CA | 158.50±43.80 | 325.21±109.35* | 342.05±97.09* | 419.67±121.84** | 490.99±129.20** |
| P-UDCA | 22.16±7.71 | 60.44±8.75** | 59.13±11.07** | 72.94±14.58** | 189.47±54.38** |
| P-CDCA | 50.08±7.33 | 109.92±19.08** | 276.10±71.49** | 368.08±42.90** | 1035.98±43.16** |
| P-DCA | 30.91±11.22 | 38.15±12.62 | 60.64±4.08* | 76.55±6.83** | 79.63±12.29** |
| P-LCA | 0.34±0.12 | 0.15±0.06* | 0.15±0.04** | 0.11±0.04** | 0.18±0.09* |
| P-GCA | 5.47±2.52 | 31.96±6.80** | 36.35±9.36** | 29.35±10.13** | 35.88±6.09** |
| P-GUDCA | 3.04±2.12 | 8.35±1.81** | 4.04±1.02 | 3.75±1.97 | 5.10±2.02 |
| P-GCDCA | 1.91±0.37 | 1.94±0.87 | 1.65±0.44 | 2.46±1.05 | 2.00±1.17 |
| P-GDCA | 0.31±0.12 | 0.22±0.05 | 0.28±0.07 | 0.23±0.10 | 0.19±0.06 |
| P-GLCA | 0.61±0.20 | 0.15±0.05** | 0.15±0.07** | 0.13±0.05** | 0.14±0.07** |
| P-TCA | 123.55±41.33 | 267.44±97.88* | 334.35±71.95** | 364.49±56.17** | 480.73±92.59** |
| P-TCDCA | 51.04±11.27 | 78.35±18.98* | 105.51±18.33** | 40.45±19.49 | 65.26±9.67* |
| P-TLCA | 7.77±2.42 | 2.62±1.65** | 3.04±1.73** | 1.69±0.91** | 1.87±0.70** |
| P-TDCA | 124.15±11.92 | 152.92±18.35* | 148.36±23.90* | 157.83±33.93* | 180.00±25.99* |
| P-TUDCA | 102.48±20.62 | 116.17±12.30 | 122.58±32.58 | 112.04±24.24 | 141.18±33.15* |
| L-CA | 97.00±6.58 | 243.16±53.76** | 265.04±44.33** | 393.81±88.39** | 493.95±133.45** |
| L-UDCA | 128.84±11.50 | 60.36±11.96** | 46.94±6.55** | 49.76±10.97** | 38.62±7.27** |
| L-CDCA | 17.72±8.21 | 64.10±9.79** | 68.48±8.16** | 75.92±4.10** | 94.89±14.16** |
| L-DCA | 29.34±10.86 | 62.45±9.75* | 67.46±6.44* | 69.05±13.11* | 86.31±5.20** |
| L-LCA | 0.06±0.01 | 0.04±0.01* | 0.05±0.01 | 0.04±0.01* | 0.03±0.01* |
| L-GCA | 23.15±6.49 | 38.63±14.92 | 56.42±8.88** | 55.04±10.57** | 96.12±11.90** |
| L-GUDCA | 9.01±1.95 | 6.00±2.61 | 6.93±1.15 | 7.06±1.95 | 16.09±1.99** |
| L-GCDCA | 1.95±0.49 | 1.22±0.38* | 2.10±0.48 | 1.74±0.90 | 3.89±1.25* |
| L-GDCA | 6.28±2.43 | 4.49±1.57 | 8.36±3.03 | 10.48±2.76 | 12.06±5.85 |

| | | | | | |
|---|---|---|---|---|---|
| L-GLCA | 7.56±0.87 | 3.32±0.41** | 3.12±0.75** | 3.46±0.82** | 3.64±1.20** |
| L-TCA | 109.33±28.92 | 175.25±48.84* | 334.35±71.95** | 381.07±72.71** | 471.05±52.60** |
| L-TCDCA | 41.04±10.49 | 70.52±8.06** | 94.13±17.65** | 64.81±9.13** | 69.54±6.65** |
| L-TLCA | 6.14±1.72 | 1.93±0.62** | 2.40±1.05** | 1.31±0.36** | 1.99±0.70** |
| L-TDCA | 168.30±35.93 | 113.42±24.66 | 137.83±10.80 | 238.65±18.37* | 218.01±25.42 |
| L-TUDCA | 117.74±14.87 | 88.52±22.58 | 106.42±10.79 | 85.36±17.17* | 158.86±27.94* |
| I-CA | 78.25±14.33 | 197.76±20.32** | 178.73±11.93** | 175.17±31.53** | 143.30±20.94** |
| I-UDCA | 207.47±24.44 | 42.97±11.10** | 24.50±7.86** | 14.07±8.08** | 14.60±7.05** |
| I-CDCA | 7.20±2.92 | 31.64±4.73** | 23.70±8.31** | 21.69±6.92** | 13.40±3.06* |
| I-DCA | 27.04±4.15 | 55.18±6.53** | 52.39±10.14** | 42.69±8.83* | 21.73±7.51 |
| I-LCA | 10.06±1.22 | 11.63±2.44 | 6.75±1.40** | 6.20±1.39** | 5.64±1.92** |
| I-GCA | 7.83±6.51 | 23.20±6.14** | 16.16±3.84 | 25.12±6.17** | 23.33±6.10** |
| I-GUDCA | 4.90±0.97 | 2.94±1.07* | 1.71±0.41** | 1.09±1.13** | 0.58±0.76** |
| I-GCDCA | 17.91±5.60 | 47.36±9.72** | 39.12±2.36* | 29.82±6.34* | 29.76±5.23* |
| I-GDCA | 5.77±3.50 | 16.33±2.06** | 8.58±1.68 | 13.13±1.38** | 13.67±3.10** |
| I-GLCA | 1.64±0.33 | 1.46±0.41 | 1.18±0.38 | 0.55±0.31** | 1.37±0.47 |
| I-TCA | 127.28±7.98 | 72.01±6.90** | 48.14±15.35** | 33.99±13.36** | 32.10±4.11** |
| I-TCDCA | 32.84±6.67 | 15.03±3.22* | 7.45±5.09** | 6.68±3.45** | 10.28±4.63** |
| I-TLCA | 86.27±8.86 | 78.98±10.31 | 46.04±16.85** | 38.84±13.99** | 49.19±7.18** |
| I-TDCA | 22.56±2.57 | 9.52±1.65** | 6.04±3.62** | 2.40±0.94** | 2.29±0.98** |
| I-TUDCA | 131.77±21.84 | 93.19±12.23* | 37.48±9.80** | 16.65±3.96** | 14.20±6.41** |

*$p < 0.05$, compared with heathy controls; **$p < 0.01$, compared with heathy controls.

**Table S4.** The classification results by BLDA.

| | Groups | Health control | Hepatitis | Cirrhosis | HCC | Advanced HCC | Total |
|---|---|---|---|---|---|---|---|
| Count | Health control | 5 | 1 | 0 | 0 | 0 | 6 |
| | Hepatitis | 0 | 6 | 0 | 0 | 0 | 6 |
| | Cirrhosis | 0 | 1 | 4 | 1 | 0 | 6 |
| | HCC | 0 | 0 | 1 | 5 | 0 | 6 |
| | Advanced cancer | 0 | 0 | 0 | 0 | 6 | 6 |
| % | Health control | 83.3 | 16.7 | 0 | 0 | 0 | 100 |
| | Hepatitis | 0 | 100 | 0 | 0 | 0 | 100 |
| | Cirrhosis | 0 | 16.7 | 66.7 | 16.7 | 0 | 100 |
| | HCC | 0 | 0 | 16.7 | 83.3 | 0 | 100 |
| | Advanced cancer | 0 | 0 | 0 | 0 | 100 | 100 |

**Table S5**. 15 gene sets with FDR<25%, NES>1.5 and good significance (*P*-value<0.05) of GSEA enrichment analysis for patient samples which collected from TCGA database.

| No. | Name | gene | description | collections | source organism | contributor |
|---|---|---|---|---|---|---|
| 1 | HALLMARK_BILE_ACID_METABOLISM | 112 | Genes involve in metabolism of bile acids and salts. | H | Homo sapiens | MSigDB Team |
| 2 | GOMF_CHOLESTEROL_BINDING | 51 | Binding to cholesterol (cholest-5-en-3-beta-ol); the principal sterol of vertebrates and the precursor of many steroids, including bile acids and steroid hormones. [GOC:jl, ISBN:0198506732] | C5 GO | Homo sapiens | Gene Ontology Consortium |
| 3 | GOBP_BILE_ACID_BIOSYNTHETIC_PROCESS | 36 | The chemical reactions and pathways resulting in the formation of bile acids, any of a group of steroid carboxylic acids occurring in bile. [GOC:go_curators] | C5 GO | Homo sapiens | Gene Ontology Consortium |
| 4 | GOBP_BILE_ACID_METABOLIC_PROCESS | 49 | The chemical reactions and pathways involving bile acids, a group of steroid carboxylic acids occurring in bile, where they are present as the sodium salts of their amides with glycine or taurine. [GOC:go_curators] | C5 GO | Homo sapiens | Gene Ontology Consortium |
| 5 | GOBP_BILE_ACID_AND_BILE_SALT_TRANSPORT | 31 | The directed movement of bile acid and bile salts into, out of or within a cell, or between cells, by means of some agent such as a transporter or pore. [GOC:dph, GOC:krc, PMID:12663868, PMID:14699511] | C5 GO | Homo sapiens | Gene Ontology Consortium |
| 6 | GOBP_REGULATION_OF_CHOLESTEROL_METABOLIC_PROCESS | 40 | Any process that modulates the rate, frequency, or extent of cholesterol metabolism, the chemical reactions and pathways involving cholesterol, cholest-5-en-3 beta-ol, the principal sterol of vertebrates and the precursor of many steroids, including bile acids and steroid hormones. [GOC:BHF, GOC:dph, GOC:tb] | C5 GO | Homo sapiens | Gene Ontology Consortium |
| 7 | REACTOME_SYNTHESIS_OF_BILE_ACIDS_AND_BILD_BILE_SALTS_VIA_7ALPHA_HYDROXYCHOLESTEROL | 24 | Synthesis of bile acids and bile salts via 7alpha-hydroxycholesterol | C2 CP | Homo sapiens | Reactome |
| 8 | REACTOME_SYNTHESIS_OF_BILE_ACIDS_AND_BILD_BILE_SALTS_VIA_27_HYDROXYCHOLESTEROL | 15 | Synthesis of bile acids and bile salts via 27-hydroxycholesterol | C2 CP | Homo sapiens | Reactome |
| 9 | REACTOME_SYNTHESIS_OF_BILE_ACIDS_AND_BILD_BILE_SALTS | 34 | Synthesis of bile acids and bile salts | C2 CP | Homo sapiens | Reactome |
| 10 | WP_FARNESOID_X_RECEPTOR_PATHWAY | 19 | Farnesoid X receptor pathway | C2 CP | Homo sapiens | WikiPathways |
| 11 | KEGG_PRIMARY_BILE_ACID_BIOSYNTHESIS | 16 | Primary bile acid biosynthesis | C2 CP | Homo sapiens | Kyoto Encyclopedia of Genes and Genomes |
| 12 | REACTOME_CYTOCHROME_P450_ARRANGED_BY_SUBY_SUBSTRATE_TYPE | 64 | Cytochrome P450 - arranged by substrate type | C2 CP | Homo sapiens | Reactome |
| 13 | WP_DRUG_INDUCTION_OF_BILE_ACID_PATHWAY | 17 | Drug induction of bile acid pathway | C2 CP | Homo sapiens | WikiPathways |
| 14 | REACTOME_BILE_ACID_AND_BIL | 45 | Bile acid and bile salt metabolism | C2 CP | Homo sapiens | Reactome |

| | | | | | | |
|---|---|---|---|---|---|---|
| | E_SALT_METABOLISM | | | | | |
| 15 | REACTOME_RECYCLING_OF_BIL E_ACIDS_AND_SALTS | 18 | Recycling of bile acids and salts | C2 CP | Homo sapiens | Reactome |

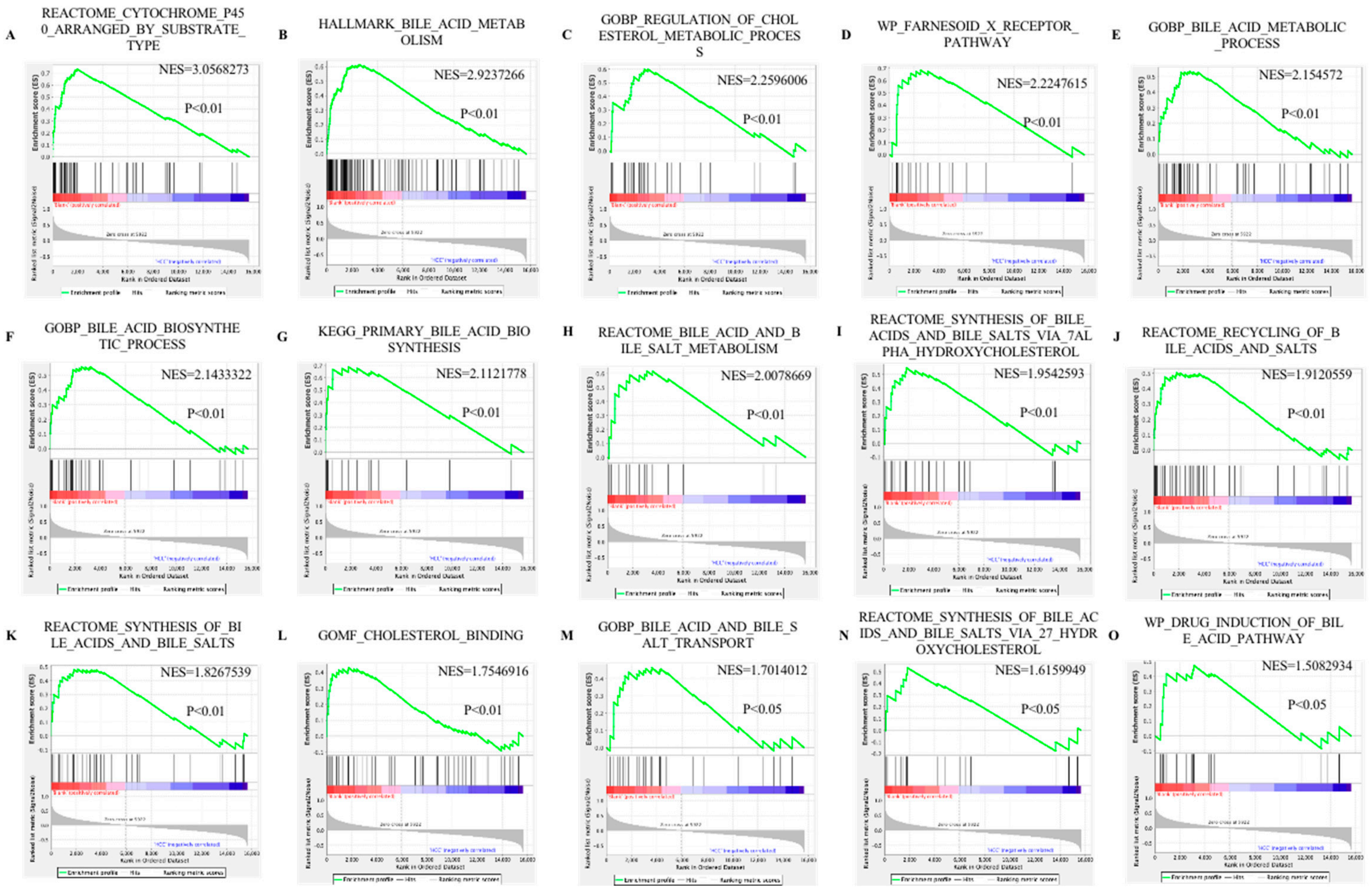


**Figure S1.** 15 gene sets with FDR<25%, NES>1.5 and good significance (*P*-value<0.05) of GSEA enrichment analysis for patient samples which collected from TCGA database.The order of the gene sets is arranged according to NES value (A-O).

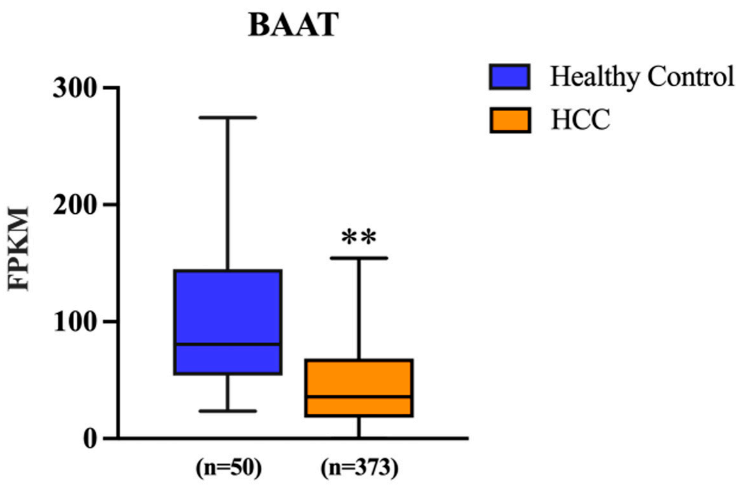


**Figure S2.** BAAT expression is generally downregulated in human HCC tissues. Expression of BAAT in patients with HCC based on data from TCGA Liver Hepatocellular Carcinoma dataset. FPKM, Fragments Per Kilobase of exon model per Million mapped fragments. Figure indicates statistical significance compared with heathy controls by t-tests, $^{**}p < 0.01$.
